# Prediction of Acoustic Fields Using a Lattice-Boltzmann Method and Deep Learning

**DOI:** 10.1007/978-3-030-59851-8_6

**Published:** 2020-09-15

**Authors:** Mario Rüttgers, Seong-Ryong Koh, Jenia Jitsev, Wolfgang Schröder, Andreas Lintermann

**Affiliations:** 8 University of Tennessee at Knoxville, Knowville, TN USA; 9 Department of Mathematics, KIT für Technologie Karlsruhe, Karlsruhe, Baden-Württemberg Germany; 10 Computational Science, Helmholtz-Zentrum Dresden Rossendorf, Dresden, Sachsen Germany; 11 Extreme Computing Research Center, King Abdullah University of Science and Technology, Thuwal, Saudi Arabia; 12 Institute of Aerodynamics and Chair of Fluid Mechanics, RWTH Aachen University, Wüllnerstraße 5a, 52062 Aachen, Germany; 13 Jülich Supercomputing Centre, Forschungszentrum Jülich GmbH, Wilhelm-Johnen-Straße, 52425 Jülich, Germany; 14 Jülich Aachen Research Alliance Center for Simulation and Data Science, Seffenter Weg 23, 52074 Aachen, Germany

**Keywords:** Deep convolutional neural networks, Aeroacoustic predictions, Lattice-boltzmann method

## Abstract

Using traditional computational fluid dynamics and aeroacoustics methods, the accurate simulation of aeroacoustic sources requires high compute resources to resolve all necessary physical phenomena. In contrast, once trained, artificial neural networks such as deep encoder-decoder convolutional networks allow to predict aeroacoustics at lower cost and, depending on the quality of the employed network, also at high accuracy. The architecture for such a neural network is developed to predict the sound pressure level in a 2D square domain. It is trained by numerical results from up to 20,000 GPU-based lattice-Boltzmann simulations that include randomly distributed rectangular and circular objects, and monopole sources. Types of boundary conditions, the monopole locations, and cell distances for objects and monopoles serve as input to the network. Parameters are studied to tune the predictions and to increase their accuracy. The complexity of the setup is successively increased along three cases and the impact of the number of feature maps, the type of loss function, and the number of training data on the prediction accuracy is investigated. An optimal choice of the parameters leads to network-predicted results that are in good agreement with the simulated findings. This is corroborated by negligible differences of the sound pressure level between the simulated and the network-predicted results along characteristic lines and by small mean errors.

## Introduction

State-of-the-art machine learning (ML), e.g., deep learning (DL) techniques that require very large datasets for successful training, can greatly benefit from high-performance computing (HPC) simulations. Such simulations can be used to generate lots of training data. They come with the flexibility to obtain datasets corresponding to various task setting parameterizations, which can be used to train ML models. In contrast, obtaining data from experiments can be costly, less flexible, and sometimes even impossible. Trained ML models are capable of performing different forms of predictions on variables of interest if novel input is provided. Their knowledge is based on observations of phenomena acquired from the training on simulated data. Such data-driven models are often used as surrogate models to accelerate predictions compared to classical computationally demanding simulators, given the accuracy provided is sufficient.

Especially in the field of computational fluid dynamics (CFD), DL models trained on simulated data are capable of accelerating the prediction of flow fields. Conventional flow solvers need time to reach solutions at which the impact of initial conditions vanishes. Then, they can be used to compute, e.g., averaged results of the flow. In this case, the period of averaging needs to be bridged before the results can be analyzed. To overcome this issue, methods to accelerate the prediction of steady flow fields using convolutional neural networks (CNNs) are studied [3, 7]. In [7], the flow over simplified vehicle bodies is predicted with CNNs. The corresponding surrogate model is considerably faster than traditional flow solvers. In [3], CNNs are successfully applied to predict flow fields around airfoils with varying angles of attack and Reynolds numbers. Lee and You [16] predict the unsteady flow over a circular cylinder using DL methods. They reveal large-scale vortex dynamics to be well predictable by their models. In [17], CNNs to predict unsteady three-dimensional turbulent flows are investigated. The CNNs correctly learn to transport and integrate wave number information contained in feature maps. Additionally, a method that can optimize the number of feature maps is proposed. Unsteady flow and force coefficients are the main focus of the investigations in [22], in which a data-driven method using a CNN for model reduction of the Navier-Stokes equations is presented. In [27], a generative adversarial network (GAN) to forecast movements of typhoons is used and satellite images along with velocity information from numerical simulations are incorporated. This allows for 6-hour predictions of typhoons with an averaged error ${<}95.6$ km. Unlike numerical predictions on HPC systems, the GAN-based method takes only seconds. Bode et al. [4] propose a physics-informed GAN and successfully model flow on subgrid scales in turbulent reactive flows.

To improve quality and robustness of DL models, training is frequently performed on very large data sets obtained from simulations run on HPC systems. In aerodynamic problems, small-scale structures and/or fluid mechanics based perturbations can strongly influence the acoustic field although they might contain only a small amount of total energy. In many engineering applications, modeling flow-induced sound requires interdisciplinary knowledge about fluid mechanics, acoustics, and applied mathematics. Furthermore, the numerical analysis demands high-resolution numerical simulations to accurately determine the various flow phenomena, e.g., turbulent shear layers [24], fluid-structure interactions [6], and combustion processes [29], that determine the acoustic field. The sheer quantity and often high dimensionality of the parameters describing such flow fields complicate post-processing of the simulated data. This poses a challenge to derive new control models and to make progress in design optimizations [13, 33]. The turn-around time between prototyping and manufacturing depends on the complexity of fundamental physical mechanisms. A recent effort to enhance the efficiency of design development employs an ML framework to predict acoustic fields of a variety of fan nozzle and jet configurations [21]. Although the concept has not yet been realized, this ML-based approach illustrates a prospective possibility to reduce design cycle times of new engine configurations.

The main objective of the present study is the prediction of acoustic fields via a robust ML model based on a deep encoder-decoder CNN. The CNN is trained by acoustic fields containing noise sources surrounded by multiple objects. The numerical results are obtained from simulations using a lattice-Boltzmann (LB) method. They include the simulation of wave propagation, reflection, and scattering due to the interaction with sound-hard surfaces.

In the following, the numerical methods to predict room aeroacoustics with CNNs are described in Sect. 2. Subsequently, results from the sound fields predicted by CNNs are presented and juxtaposed to results of LB simulations in Sect. 3. Finally, a summary is given, conclusions are drawn, and an outlook is presented in Sect. 4.

## Numerical Methods

To generate training data for the CNN, aeroacoustic simulations are run with an LB method on two-dimensional rectangular meshes. The LB method is described in Sect. 2.1, followed by a presentation of the geometrical setup, and the computational meshes in Sect. 2.2. Section 2.3 explains the imposed boundary and initial conditions. Section 2.4 describes how the acoustic fields are analyzed. Finally, the network architecture for the prediction of aeroacoustic fields is presented in Sect. 2.5.

### Lattice-Boltzmann Method

To compute the aeroacoustic pressure field, an LB method is employed. The governing equation is the Boltzmann equation with the simplified right-hand side (RHS) Bhatnagar-Gross-Krook (BGK) collision term [2]

1 $$\begin{aligned} \frac{\partial f}{\partial t} + \xi _{k} \frac{\partial f}{\partial x_{k}} = - \frac{1}{\tau }(f-f^{eq}). \end{aligned}$$

The particle probability density functions (PPDFs) $f=f(\vec {x},\vec {\xi },t)$ describe the probability to find a particle of a fluid around a location $\vec {x}$ with a particle velocity $\vec {\xi }$ at time *t* [1, 8]. The left-hand side (LHS) of Eq. (1) describes the evolution of fluid particles in space and time, while the RHS describes the collision of particles. The collision process is governed by the relaxation parameter $1/\tau$ with relaxation time $\tau$ to reach the Maxwellian equilibrium state $f^{eq}$. The discretized form of Eq. (1) yields the lattice-BGK equation

2 $$\begin{aligned} f_{k}(\vec {x}+\xi _{k}\varDelta t, t+\varDelta t) = f_{k}(\vec {x},t)- \frac{1}{\tau }(f_{k}(\vec {x},t)-f_{k}^{eq}(\vec {x},t)). \end{aligned}$$

The quantity $\varDelta t$ is the time increment and $\tau$ is a function of the kinematic viscosity $\nu$ and the speed of sound $c_{s}$, i.e.,

3 $$\begin{aligned} \tau = \frac{\nu +\varDelta t c_s^2/2}{c_s^2}. \end{aligned}$$

In the LB context, the spatial and temporal spacing are set to $\varDelta x=\varDelta t=1.0$ such that $c_{s}=1/\sqrt{3}$. Table 1 exemplarily lists the LB viscosity for two meshes $\mathcal {M}_c$ and $\mathcal {M}_f$ with different resolutions. Note that these values are derived in Sect. 2.3. The LB viscosity is an artificial parameter simply influencing the time step, i.e., how much physical time $\tilde{t}$ is covered by a single $\varDelta t$ in the simulation. Using the viscosities listed in Table 1 would lead to extremely small time steps. For this reason and in order to conduct numerically stable simulations, $\nu$ is set to a feasible value according to [28]. The indices *k* in Eq. (2) depend on the discretization scheme and represent the different directions of the PPDFs. In this work, the two-dimensional discretization scheme with 9 PPDFS, i.e., the D2Q9 model [25] is used. The discretized equilibrium PPDF is given by

4 $$\begin{aligned} f_{k}^{eq}=w_k \rho \left( 1+\frac{\xi _k \vec {u}}{c_s^2}+\frac{(\xi _k\vec {u})^2}{2c_s^4}-\frac{\vec {u}^2}{2c_s^2}\right) , \end{aligned}$$

where the quantities $w_k$ are weighting factors for the D2Q9 scheme given by 4/9 for $k\in \{0\}$, 1/9 for $k\in \{1,\ldots ,4\}$, and 1/36 for $k\in \{5,\ldots ,8\}$, and $\vec {u}$ is the fluid velocity. The macroscopic variables can be obtained from the moments of the PPDFs, i.e., the density $\rho = \sum _{k}{f_k}$. The pressure can be computed using the ideal gas law by $p=c_s^2\rho =(1/3)\rho$.

**Table 1.** Physical quantities of the setup and the non-dimensional viscosity $\nu$.

Mesh	$\varDelta \tilde{x}~[m]$	$\varDelta \tilde{t}~[s]$	$\tilde{\omega }~[Hz]$	$\tilde{\nu }~[m^2/s]$	$\nu$
$\mathcal {M}_c$	0.2	$3.4 \cdot 10^{-4}$	58.8	$1.551717\cdot 10^{-5}$	$1.318959 \cdot 10^{-7}$
$\mathcal {M}_f$	0.1	$1.7 \cdot 10^{-4}$	117.6	$1.551717\cdot 10^{-5}$	$2.637920 \cdot 10^{-7}$

The LB method has been chosen for several reasons [18]: (i) the computations can be performed efficiently in parallel, (ii) it is straightforward to parallelize the code, (iii) boundary conditions can easily be applied in contrast to, e.g., cut-cell methods, and (iv) there is no need to solve a pressure Poisson-equation for quasi-incompressible flow as the pressure and hence the acoustic field is an explicit result of the lattice-BGK algorithm. Furthermore, the LB method can be applied for low to high Knudsen numbers *Kn*. In the continuum limit, i.e. for small *Kn*, the Navier-Stokes and Euler equations can directly be derived from the Boltzmann equation and the BGK model [8].

**Fig. 1.**
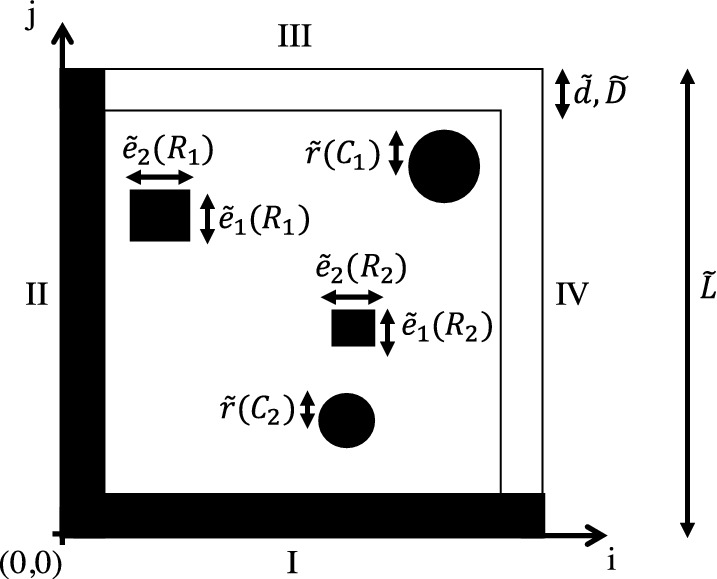
Computational domain.

### Geometrical Setup and Computational Meshes

The computational domain has a square shape containing randomly distributed objects. In physical space, denoted in the following by , the domain has an edge length of $\tilde{L}=25.6$ m. Throughout this study, the number of objects varies depending on the complexity of a computation. The domain of the most complex case is shown in Fig. 1. It has two rectangular objects $R_1$ and $R_2$ and two circular objects $C_1$ and $C_2$. Their size is a function of the characteristic length $\tilde{C}=\tilde{L}/16$, i.e., $R_1$ and $R_2$ have edge lengths $\tilde{e}_1(R_1), \tilde{e}_2(R_1),\tilde{e}_1(R_2),\tilde{e}_2(R_2)\in [\tilde{C},2\tilde{C}]$, and $C_1$ and $C_2$ have radii $\tilde{r}(C_1),\tilde{r}(C_2)\in [\tilde{C},2\tilde{C}]$. All objects have a minimum distance of $\tilde{d}=\tilde{C}$ from the domain boundaries and may overlap.

Two-dimensional uniformly refined meshes $\mathcal {M}_f$ and $\mathcal {M}_c$ with two distinct resolutions are generated in Cartesian coordinates. In the fine mesh $\mathcal {M}_f$ each cell has an edge length of $\varDelta \tilde{x}_f = (1/16)\tilde{C}=0.1$ m resulting in $256\times 256$ cells. The coarse mesh $\mathcal {M}_c$ has a cell length of $\varDelta \tilde{x}_c=(1/8)\tilde{C}=0.2$ m and a total of $128 \times 128$ cells.

### Boundary and Initial Conditions

Two types of boundary conditions are imposed at the four domain boundaries according to [11], i.e., non-reflecting (NRBCs) and wall boundary conditions (WBCs) are prescribed. As shown for boundaries III and IV in Fig. 1, the NRBCs have a buffer layer thickness of $\tilde{D}=\tilde{C}$ to ensure a complete dissipation of acoustic waves and to avoid reflective phenomena at the domain boundaries. In the buffer layer, an absorption term [11]

5 $$\begin{aligned} F_{ad}=-\sigma (f_{k}^{eq}(\vec {x},t)-f_{a}), \end{aligned}$$

with weighting factor $f_{a}$ and $\sigma = \sigma _{m}(\tilde{\delta }/\tilde{D})^2$ is added to Eq. (2). The quantity $\tilde{\delta }$ is the distance to the buffer layer and $\sigma _{m}$ is a constant specified as 0.1.

The WBCs are characterized by a no-slip behavior, where the PPDFs are reflectively bounced back. They are imposed as a layer with thickness $\tilde{D}=\tilde{C}$ as shown for boundaries I and II in Fig. 1, i.e., the computational domain is reduced by this thickness. In computations with WBC, a maximum number of three domain boundaries is specified as WBC in a random process. To prevent strong overlaps of acoustic waves, which may cause numerical instabilities, at least at one domain boundary an NRBC is imposed.

The acoustic fields, which are exploited to train the CNN model, are configured by a simple source *S* defined by a sinusoidal function given by

6 $$\begin{aligned} S(t) = A\cdot \sin (2 \pi \omega t), \end{aligned}$$

with a frequency $\omega =0.02\cdot (1/\varDelta t)$ and the amplitude $A=0.1\cdot \rho _\infty$ and $\rho _\infty =1.0$ in the LB context. A set of the training data is generated by the computational domains with a noise source restricted by a geometry, i.e., the minimum distance $\tilde{C}$ between the noise source and the sound-hard objects satisfies the condition $L < 2\tilde{C}$ where *L* is a distance between monopoles and domain boundaries. With $\omega = 1/T$, this yields a non-dimensionalized harmonic period of $T = 50\varDelta t$. One wavelength $\lambda$ is computed from $\lambda =u_{w}/\omega$, with $u_{w}=\varDelta x / \varDelta t$ being the velocity with which information is transported in the LB context. This results in $\lambda =50 \varDelta x$ for computations in this study, if not stated otherwise.

The relationship between $\omega$ in LB context and the frequency $\tilde{\omega }$ in physical space is obtained by inserting

7 $$\begin{aligned} \varDelta \tilde{t} = \varDelta \tilde{x} \frac{c_{s}}{\tilde{c}_{s}}, \end{aligned}$$

with the physical speed of sound $\tilde{c}_s=340$ m/s at reference temperature $T_\infty =298.15K$ into the equation for the frequency $\tilde{\omega }=0.02(1/\varDelta \tilde{t})$. The relationship between $\nu$ in the LB context and the kinematic viscosity $\tilde{\nu }$ in physical space is given by

8 $$\begin{aligned} \nu = \tilde{\nu } \cdot \frac{\varDelta \tilde{t}}{(\varDelta \tilde{x})^2}\qquad \text { with}\qquad \tilde{\nu } = \frac{1}{\tilde{\rho }_\infty }\cdot \frac{K_{1}\cdot T_\infty ^{\frac{3}{2}}}{T_\infty +K_{2}}. \end{aligned}$$

The latter equation is Sutherland’s law [32] with $\tilde{\rho }_\infty =1.184\, \mathrm{kg/m}^{3}$, $K_{1}=1.458\cdot 10^{-6}\, \mathrm{kg/(ms}\cdot \mathrm{K}^{1/2})$, and $K_{2}=110.4K$. Table 1 lists all necessary variables in their dimensional and non-dimensional form for $\mathcal {M}_f$ and $\mathcal {M}_c$.

### Evaluation of Acoustic Fields

The acoustic fields are determined by a set of the computational domains which include at least one noise source and randomized solid surfaces. For fluid cells at location $(i,j), i,j\in \{1,\ldots ,m\}$, the sound pressure level *SPL* is defined by

9 $$\begin{aligned} SPL(i,j) = 20 \log _{10}(p'_\mathrm{rms}(i,j)) , \end{aligned}$$

where the maximum number of mesh points *m* is $m=128$ for the coarse grid and $m=256$ for the fine grid configurations. The root-mean-square (rms) values of pressure fluctuations $p'$ are calculated by

10 $$\begin{aligned} p'_\mathrm{rms}(i,j)=\sqrt{\frac{\sum _{n=1}^{N}(p_{n}(i,j)-p_\mathrm{avg}(i,j))^2}{N}} , \end{aligned}$$

where $p_\mathrm{avg}(i,j)$ is the mean pressure averaged over the time period *N*, and $p_{n}(i,j)$ is the instantaneous pressure resulting from the simulation at a time step *n* within that period. Simulations are carried out for 3, 000 time steps. The averaging period $N=2,000$ starts after 1, 000 time steps when the acoustic field is fully developed.

### Machine Learning Techniques

An encoder-decoder CNN is trained to predict the *SPL* in a supervised manner using results of the aforementioned LB simulations. The CNN is fed with four types of input data:

(i) types of boundary condition;
(ii) location of monopoles;
(iii) cell distances for objects;
(iv) cell distances for monopoles.

To correctly predict aeroacoustic fields, the CNN needs to learn the impact of the various boundary conditions and the location of monopoles on the acoustic field. Therefore, considering inputs (i) and (ii), cells at location (*i*, *j*) are assigned segmentation values

11 $$\begin{aligned} \varUpsilon (i,j) = {\left\{ \begin{array}{ll} 0, &{} \text {empty or NRBC cell}\\ \frac{1}{2}, &{} \text {WBC or object cell}\\ 1, &{} \text {monopole cell} \end{array}\right. }. \end{aligned}$$

A sensitivity analysis of the input data has been performed before the training. This analysis revealed that solely using boundary parameters leads to poor predictions of the network, i.e., it is not effective for CNNs learning from flow simulations. This is in line with findings in [7]. Since acoustic signals propagate with a certain wavelength and amplitude at a certain sound speed, distances are also important parameters for learning. For this purpose, inputs (iii) and (iv) are provided to the CNN in the form of distance functions $\varPhi _o$ for objects and $\varPhi _m$ for monopoles. Such an approach has previously been used for CNNs to predict steady-state flow fields [3, 7]. The distance functions are defined by

12 $$\begin{aligned} \varPhi _{o}(\mathbf{x} )= {\left\{ \begin{array}{ll} d(\mathbf{x} ,\partial \varOmega ) &{} \mathbf{x} \notin \varOmega \\ 0 &{} \mathbf{x} \in \{\partial \varOmega ,\varOmega \} \end{array}\right. }&\qquad \text { and }\qquad \varPhi _{m}(\mathbf{x} )=d(\mathbf{x} ,M), \end{aligned}$$

i.e., for each cell $\mathbf{x}$ with location (*i*, *j*) in a domain the minimal distances $d(\mathbf{x} ,\partial \varOmega )$ and $d(\mathbf{x} ,M)$ to the boundary $\partial \varOmega$ of an object $\varOmega$ and to a monopole *M* are determined. Obviously, it is $\varPhi _o(\mathbf{x} )=\varPhi _m(\mathbf{x} )=0$ on the boundary and exactly at the monopole source. For $\varPhi _o$, an assignment of negative distances for cells inside of an object, as it is usually used by signed-distance functions, turned out to have a negative impact on predictions, which is why $\forall (\mathbf{x} \in \varOmega :\varPhi _o=0)$. The distances are computed by the fast marching method [30] and are normalized by $\tilde{L}$. Learning from distances like inputs (iii) and (iv) alone results in mispredictions near domain boundaries. A combination of all presented types of inputs has been found to favorably affect predictions.

**Fig. 2.**
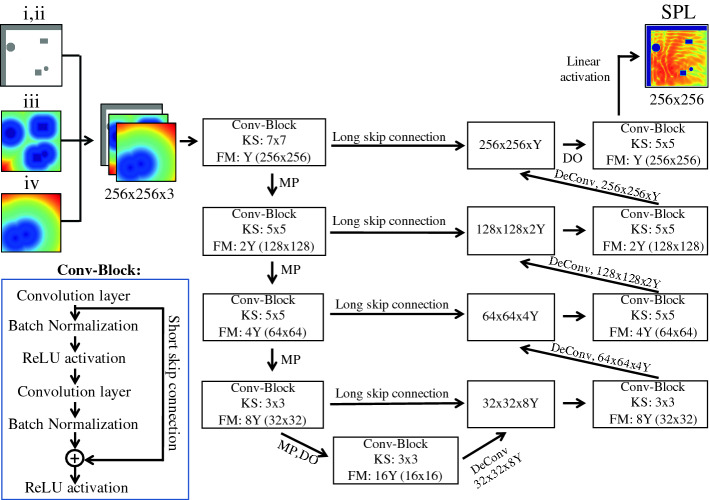
Network architecture of the AFP including size and number of feature maps (FMs) as a multiple of *Y*, kernel size (KS), $2\,\times \,2$ maximum pooling layers (MP), dropout layers (DO), convolutional blocks, and deconvolutional layers.

In the following, the CNN used for predicting the *SPL* fields is referred to as acoustics field predictor (AFP). The corresponding network architecture is shown in Fig. 2 for a case that uses arrays with the size of $\mathcal {M}_f$ as inputs. Inputs (i) and (ii) are combined to one array. Together with fields (iii) and (iv) they are stacked to form channels of the input. It should be noted that physical quantities such as the pressure distribution are a solution of the acoustic fields computation and constitute the ground truth. They are not known a priori and hence cannot be used for training.

The architecture is inspired by distinct architectures that employ long skip connections between encoder and decoder layers [19, 26, 34], like for instance U-net architectures, which have been successfully used for medical image segmentation [26]. Skip connections between encoding and decoding paths allow the re-use and fusion of features on different scales. To preserve information from features on all scales, the activity of each encoder layer is directly fed to the corresponding decoder layer via long skip connections. These connections are chosen to have residual form, adding the activity of encoder layers to the output of decoder layers. This setup is similar to [19], however, different from the original U-net architecture, where long skip connections have dense form and concatenate layers on the same scale. As depicted in Fig. 2, the residual long skip connections perform identity mapping by adding source encoder layer outputs to target decoder layer outputs [9, 19]. This kind of connectivity allows for direct gradient flow from higher to lower layers across all hierarchy stages during the backward pass, which prevents common issues with vanishing gradients in deep architectures. In contrast to dense long skip connections, residual skip connections lead to smaller numbers of activations to be handled in the decoding path during the forward and backward passes. As a consequence, they decreased memory consumption and are more efficient and faster in training without sacrificing prediction accuracy. Short skip residual connections are also used in so called convolutional residual blocks (Conv-Blocks). Here, convolutional layers, batch normalization (BN), and rectified linear unit (ReLU) activation functions are employed. BN acts as a regularizer, shifting activity of the layers to zero mean, unit variance. This leads to faster and more reliable network convergence [10]. The number of feature maps (FMs) is a multiple of a given factor *Y*. The output of the first convolutional layer is added to the input of the last ReLU activation, see Fig. 2, which defines residual short skip connections in Conv-Blocks. A combination of long and short skip connections leads to faster convergence and stronger loss reduction [5]. In the encoder path, downscaling is performed by $2\times 2$ maximum pooling layers (MP). To further avoid overfitting, yet another regularization method, dropout (DO) [31] is used during training, with a DO probability of $P=0.5$. The final layer is fully connected with a linear activation function, which is frequently used for regression outputs [15]. Weights and biases are initialized from a truncated normal distribution centered around the origin with a standard deviation of $\sigma _{std}=\sqrt{2/f}$, where *f* is the number of connections at a layer [9]. They are updated by an adaptive moments (ADAM) optimizer [12]. The ADAM optimizer adjusts the learning rate (LR) by considering an exponentially decaying average of gradients computed in previous update steps. The initial learning rate is set to $LR=0.001$. The batch size *BS* represents the number of training data passed to the network in a single training iteration. In Sect. 3 it will be shown that in this context a batch size of $BS=5$ achieves the best results. Therefore, it is used throughout this study, if not stated otherwise. The ground truth *GT* distribution $SPL_{GT}$ is obtained from

13 $$\begin{aligned} SPL_{GT} = \frac{SPL-SPL_{mean}}{SPL_{std}} , \end{aligned}$$

where $SPL_{mean}$ and $SPL_{std}$ are the mean and the standard deviation of the complete training dataset of the a priori simulations. The predictions need to be denormalized before the SPL can be analyzed.

Data augmentation is used to increase training data diversity and to encourage learning of useful invariances. Therefore, the coordinate axes *i* and *j* are transposed randomly. Furthermore, for inputs (i) and (ii), the segmentation values $\varUpsilon (i,j)$ are changed to augmented inputs $\varUpsilon _{augm}(i,j)$ according to

14 $$\begin{aligned} \varUpsilon _{augm}(i,j)= {\left\{ \begin{array}{ll} \varUpsilon (i,j) &{} i=j \\ \varUpsilon (j,i) &{} i\ne j. \end{array}\right. } \end{aligned}$$

The total loss $L_{tot}$ between simulated (superscript “*sim*”) and predicted (superscript “*pred*”) *SPL* values is defined by

15 $$\begin{aligned} L_{tot}=L_{MSE} + 2\cdot \underbrace{\left( L_{GDL}^{I} + L_{GDL}^{II} + L_{GDL}^{III} + L_{GDL}^{IV}\right) }_{L_{GDL}}, \end{aligned}$$

which is a combination of the mean squared error *MSE*

16 $$\begin{aligned} L_{MSE}=\frac{1}{m^{2}}\sum _{i=1}^{m}\sum _{j=1}^{m}\left( SPL{(i,j)^{sim}}-SPL{(i,j)^{pred}}\right) ^2 \end{aligned}$$

with $1\le i,j\le m$ and a gradient difference loss $L_{GDL}$. Gradient losses *GDL* in *i*- and *j*-directions are considered by $L_{GDL_{I}}$ and $L_{GDL_{II}}$, and diagonal gradients by $L_{GDL_{III}}$ and $L_{GDL_{IV}}$.

Three types of gradient losses are addressed in this work. The four directions indicated by roman numbers *I*–*IV* in Eq. (15) are defined by introducing integer variables *k* and *l*, i.e., the four directions are denoted by $I:(k=1,l=1)$, $II:(k=2,l=2)$, $III:(k=1,l=2)$, and $IV:(k=2,l=1)$. In the first type, $L_{GDL_{A}}$, the difference between two neighboring cells is considered, inspired by the gradient loss in the work of Mathieu et al. [20]

17 $$\begin{aligned} L_{GDL_{A}} =\,&\frac{1}{(m-1)(m-\mathrm {mod}(p,2))} \cdot \nonumber \\ \sum _{i}\sum _{j}&\big [ SPL_{i+s,j+t}^{sim} - SPL_{i,j}^{sim} - SPL_{i+s,j+t}^{pred} + SPL_{i,j}^{pred} \big ]^2 . \end{aligned}$$

In Eq. (17) the gradient losses of four neighboring points are defined by the notations $p=\mathrm {mod}(k,2)+\mathrm {mod}(l,2)$, $s=1-\mathrm {mod}(k+1,2)\cdot \mathrm {mod}(l+1,2)$, and $t=(-1)^{k+1}\cdot \mathrm {mod}(p,2)+1-s$. The gradient loss terms of the first type have a 1st-order accuracy in terms of a forward difference (FD) formulation [23]. To integrate radial propagation of a point source into the loss function, central difference (CD) schemes are added. The gradient loss $L_{GDL_{B}}$ uses a 2nd-order accurate CD formulation that incorporates two neighboring cells. The 2nd-order accurate gradient loss terms in a two-dimensional domain read

18 $$\begin{aligned} L_{GDL_{B}} =\,&\frac{1}{4~\mathrm {mod}(k+l+1,2)+8~\mathrm {mod}(k+l,2)(m-2)(m-2\cdot \mathrm {mod}(p,2))} \cdot \nonumber \\ \sum _{i}\sum _{j}&\big [SPL_{i+s,j+t}^{sim} - SPL_{i-s,j-t}^{sim} - SPL_{i+s,j+t}^{pred} + SPL_{i-s,j-t}^{pred} \big ]^2 \end{aligned}$$

The third type of gradient loss, $L_{GDL_{C}}$, is formulated with a 4th-order accurate CD scheme and includes four neighboring cells, i.e., two cells in each direction

19 $$\begin{aligned} L_{GDL_{C}} =\,&\frac{1}{144~\mathrm {mod}(k+l+1,2)+32~\mathrm {mod}(k+l,2)(m-4)(m-4\cdot \mathrm {mod}(p,2))} \cdot \nonumber \\ \sum _{i}\sum _{j}&\big [-SPL_{i+2s,j+2t}^{sim} + 8 SPL_{i+s,j+t}^{sim} - 8 SPL_{i-s,j-t}^{sim} + SPL_{i-2s,j-2t}^{sim} \nonumber \\&+ SPL_{i+2s,j+2t}^{pred} - 8 SPL_{i+s,j+t}^{pred} + 8 SPL_{i-s,j-t}^{pred} - SPL_{i-2s,j-2t}^{pred} \big ]^2 . \end{aligned}$$

The cell-wise prediction accuracy is evaluated by the absolute error

20 $$\begin{aligned} \varXi (i,j) = \frac{\left| SPL^{pred}(i,j) - SPL^{sim}(i,j)\right| }{|SPL^{sim}_{max}-SPL^{sim}_{min}|} . \end{aligned}$$

between $SPL^{pred}$ and $SPL^{sim}$ with $SPL^{sim}_{max}=\max (SPL^{sim})$ and $SPL^{sim}_{min}=\min (SPL^{sim})$. From the $\varXi$ distribution of each simulation a mean absolute error

21 $$\begin{aligned} \varGamma = \frac{1}{m^{2}}\sum _{i=1}^{m}\sum _{j=1}^{m} \varXi (i,j) \end{aligned}$$

is calculated to evaluate the prediction quality.

## Results

In the following, findings of a grid convergence study are discussed in Sect. 3.1. Results of network-predicted acoustic fields are presented for three cases 1–3 in Sects. 3.2, 3.3, and 3.4. The complexity of the cases is continuously increased.

The acoustic simulations are conducted on multiple graphics processing units (GPUs). At average, a solution on $\mathcal {M}_f$ is obtained in ${\approx } 120$ s on a single GPU. Up to ten GPUs are employed to accelerate the process. Once trained, the network predictions take only a fraction of a second on a single modern GPU and only a few seconds on any low end computer such as a laptop. For all computations the GPU partition of the JURECA system [14], Forschungszentrum Jülich, is employed. Each GPU node is equipped with two NVIDIA K80 GPUs.

**Fig. 3.**
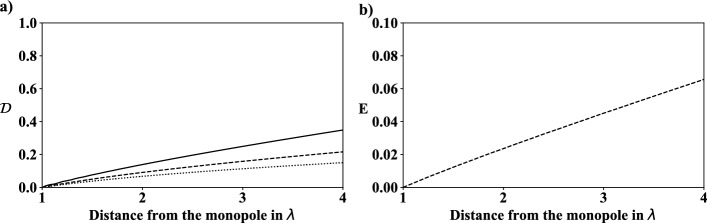
a) $\mathcal {D}=(SPL-SPL_{max})/SPL_{max}$ at a distance from up to $4\lambda$ in radial direction from a monopole placed in the center of a free field. Three resolutions for one wavelength are juxtaposed: $\mathcal {D}(\lambda =100 \varDelta x)$ $\cdot \cdot \cdot$, $\mathcal {D}(\lambda =50 \varDelta x)$ - - -, $\mathcal {D}(\lambda =25 \varDelta x)$ —. b) Error *E* between $\mathcal {D}(\lambda =50 \varDelta x$) and $\mathcal {D}(\lambda =100 \varDelta x)$.

### Grid Convergence Study

A grid convergence study is conducted in a free-field domain containing only a single monopole at the center and no walls. The impact of doubling the number of cells used to resolve one wavelength $\lambda$ on the *SPL* accuracy is investigated. Therefore, the wavelength resolutions at a distance of up to 4 wavelengths in radial direction from the source, which corresponds to the maximum appearing distance considered in the subsequently discussed cases 1–3, is analyzed. In order to obtain results in a farfield from the center for $\lambda =100 \varDelta x$, the domain is extended to $1,024 \times 1,024$ cells. Figure 3a) shows the divergence $\mathcal {D}=(SPL-SPL_{max})/SPL_{max}$ from the maximum *SPL* value $SPL_{max}$, which appears at a distance of one wavelength from the monopole location, for $\lambda =25 \varDelta x$, $50 \varDelta x$, and $100 \varDelta x$. From this figure, it is evident, that the divergence increases with increasing distance from the monopole. Furthermore, Fig. 3b) shows the error for $\lambda =50 \varDelta x$ compared to $\lambda =100 \varDelta x$, i.e., $E=\mathcal {D}(\lambda =50 \varDelta x)-\mathcal {D}(\lambda =100 \varDelta x)$. Throughout this work a wavelength of $\lambda =50\varDelta x$ is used, which covers distances up to $2\lambda$ in cases 1–2, and up to $4\lambda$ in case 3. At distances $2\lambda$ and $4\lambda$, errors of $E=0.0239$ and $E=0.0657$ are obtained. It should be noted that using $\lambda =100\varDelta x$ would massively increase the computational effort and hence, as the corresponding error is acceptable, meshes with $\lambda =50\varDelta x$ are employed in all cases.

**Table 2.** Simulation configurations defined by objects, the number of noise sources (no. noise) and simulations (no. sim) generated by randomized distributions of objects. The number of feature maps (FMs) is defined by *Y*. The gradient losses *GDL* are calculated by FD, 2nd-order-accurate, and 4th-order-accurate CD schemes. The quantities *BS* and $\varGamma$ are the batch size and the mean acoustic error.

Case	Object(s)	No. noise	No. sim	*Y*	*GDL* method	*BS*	$\varGamma$
1A	$C_{1}$	1	3,000	8	FD	5	0.17506
	$C_{1}$	1	3,000	16	FD	5	0.03312
	$C_{1}$	1	3,000	32	FD	5	0.00887
1B	$C_{1}$	1	3,000	32	FD	5	0.00887
	$C_{1}$	1	3,000	32	2nd order CD	5	0.00671
	$C_{1}$	1	3,000	32	4th order CD	5	0.00222
1C	$C_{1}$	1	3,000	32	2nd order CD	5	0.00671
	$C_{1}$	1	3,000	32	2nd order CD	10	0.00626
	$C_{1}$	1	3,000	32	2nd order CD	20	0.00413
2	$R_{1}$, $C_{1}$	1	3,000	32	2nd order CD	5	0.00359
	$R_{1}$, $C_{1}$	1	6,000	32	2nd order CD	5	0.00280
3	$R_{1}$, $R_{2}$, $C_{1}$, $C_{2}$	2	6,000	32	2nd order CD	5	0.02581
	$R_{1}$, $R_{2}$, $C_{1}$, $C_{2}$	2	10,000	32	2nd order CD	5	0.02268
	$R_{1}$, $R_{2}$, $C_{1}$, $C_{2}$	2	20,000	32	2nd order CD	5	0.01937

**Fig. 4.**
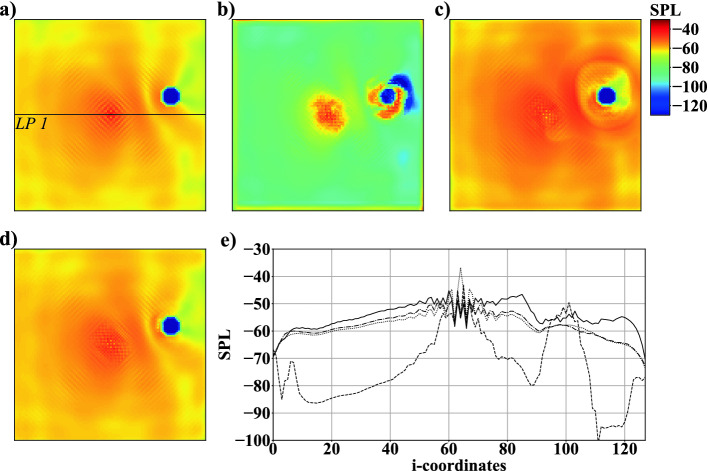
Example of *SPL* fields of case 1A: a) simulation result, b) network prediction with $Y=8$, c) $Y=16$, and d) $Y=32$; e) *SPL* distribution at $j=64$ along *LP*1: simulation result $\cdot \cdot \cdot$, network prediction with $Y=8$ - - -, $Y=16$ —, and $Y=32$ - $\cdot$ -.

### Case 1: Simple Setup and Parameter Study

The domain in case 1 contains one monopole $M_1$ at the center (8*C*, 8*C*) and one randomly positioned circular object $C_{1}$. Each computational domain consists of $128 \times 128$ cells in the two dimensions. The acoustic solutions of 3, 000 simulations are split into 2, 600 training data, 200 validation data, and 200 test data. Three sub-cases 1A, 1B, and 1C listed in Table 2 are configured by one noise source and one solid object. In case 1A, the number of FMs is investigated by varying the factor *Y* as shown in Fig. 2. Variations of $Y=8$, $Y=16$ and $Y=32$ lead to 517, 867, 2, 066, 001 and 8, 253, 089 trainable parameters. It is evident from comparing Figs. 4b), 4c), and 4d) with the simulation results in Fig. 4a) that $Y=32$ qualitatively reproduces the simulation best. For $Y=8$, the AFP completely fails to generate a physically meaningful *SPL* field. In case of $Y=16$, acoustic waves distant from the object are reproduced well, but superpositions of acoustic waves in the vicinity of the object are too strong, see Fig. 4c). The *SPL* distribution shown in Fig. 4e) along the characteristic line *LP*1, see Fig. 4a), substantiates these findings. The valley between $M_1$ and $C_1$, and the decrease of the *SPL* value in the shadow of $C_1$ are only captured well for $Y=32$. Furthermore, the CNN has problems capturing fluctuations at the center of $M_1$ as non-physical *SPL* values are found at isolated locations close to the object. The mean error $\varGamma$ listed in Table 2 shows $Y=32$ to have the lowest deviation among the three computations. The training time to reach a convergence of the loss function increased from approximately one hour for $Y=8$ up to two and four hours for $Y=16$ and $Y=32$.

**Fig. 5.**
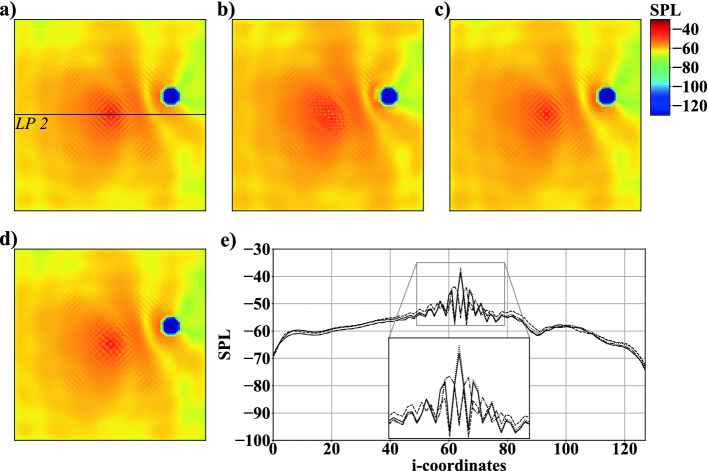
Example of SPL fields of case 1B: a) simulation result, b) network prediction with FD, c) a 2nd-order accurate CD , and d) a 4th-order accurate CD gradient loss; e) *SPL* at $j=64$ along *LP*2: simulation result $\cdot \cdot \cdot$, network prediction with FD - - -, 2nd-order accurate CD —, and 4th-order accurate CD gradient losses - $\cdot$ -.

To overcome inaccurate predictions close to monopoles, the nature of a noise source is incorporated into the loss function of the AFP. A simple FD gradient loss does not consider that monopoles are point sources spreading waves into all directions. In case 1B, two variations of losses are investigated that are based on the CD formulations provided in Sect. 2.5. From Fig. 5 it is obvious that thereby non-physical *SPL* values vanish near objects. Furthermore, Fig. 5(c) shows an improvement of the *SPL* distribution at the center and surroundings of $M_1$ predicted by a 2nd-order accurate CD gradient loss. In contrast, using a 4th-order accurate CD formulation lowers the accuracy of the predictions near $M_1$, see Fig. 5(d). It is, however, evident from Table 2 that a slightly lower $\varGamma$ is achieved than using a 2nd-order formulation. This is due to the 4th-order accurate CD gradient loss computations reproducing simulations slightly better at locations distant from monopoles and objects, see Fig. 5(e). *SPL* fluctuations at the center of $M_1$ are by far closer to the ground truth using the 2nd-order accurate formulation. Since this study focuses on the prediction of complex acoustic fields with multiple noise sources, the advantages of the 2nd-order accurate formulation are considered more valuable, i.e., in the following this type of loss is employed.

**Fig. 6.**
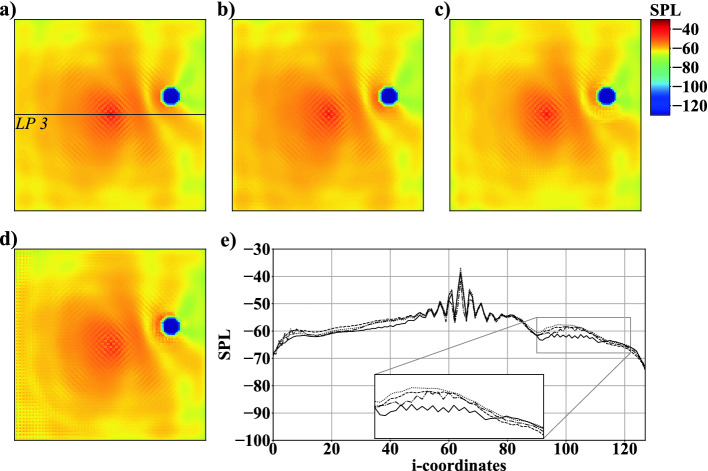
Example for *SPL* fields of case 1C: a) simulation result, b) network prediction with $BS=5$, c) $BS=10$, and d) $BS=20$; e) *SPL* distribution at $j=64$ along line *LP*3: simulation result $\cdot \cdot \cdot$, network prediction with $BS=5$ - - -, $BS=10$ —, and $BS=20$ - $\cdot$ -.

The impact of *BS* is investigated in Fig. 6. Figure 6e) plots the *SPL* distribution along line *LP*3, see Fig. 6a). Although predictions with $BS=10$ and $BS=20$ show a slight decrease of $\varGamma$, see Table 2, several shortcomings are recognizable in predicted *SPL* fields. Figures 6c) and e) show that with $BS=10$ non-physical fluctuations near the objects are introduced. These fluctuations are also present for $BS=20$ and are superimposed by inaccuracies appearing in the vicinity of $M_1$ and at the domain boundaries, i.e., $BS=5$ delivers the best results.

### Case 2: Influence of the Number of Training Data

In case 2, the number of training, validation, and test data is analyzed. Compared to case 1, the complexity is increased by adding a rectangular object $R_{1}$ to the domain. The training, validation, and test data are composed of 2, 600, 200 and 200 simulations for a total of 3, 000, and of 5, 200, 400, and 400 for a total of 6, 000 simulations. The setups for these cases are summarized in Table 2.

**Fig. 7.**
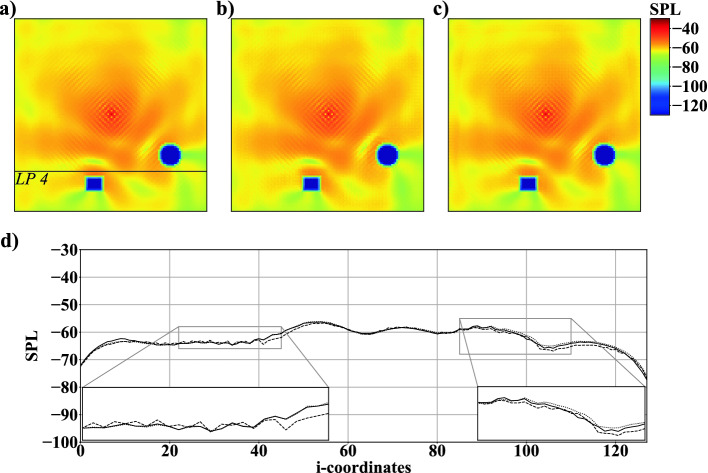
Example of *SPL* fields of case 2: a) simulation result, b) network prediction with 3, 000, and c) 6, 000 simulations; *SPL* distribution at $j=30$ along *LP*4: simulation result $\cdot \cdot \cdot$, network prediction with 3, 000 - - -, and 6, 000 simulations —.

Figure 7 compares the results of an LB simulation qualitatively and quantitatively along line *LP*4, see Fig. 7a), with predictions generated by using 3, 000 and 6, 000 simulations for learning. When the amount of data is increased, non-physical fluctuations disappear in regions, where sound waves propagate towards the surface of $R_{1}$. Furthermore, the predictions of the acoustic field in the vicinity of $C_{1}$ improve from 3, 000 to 6, 000 training datasets.

### Case 3: Complex Setup and Impact of Increasing Training Data

**Fig. 8.**
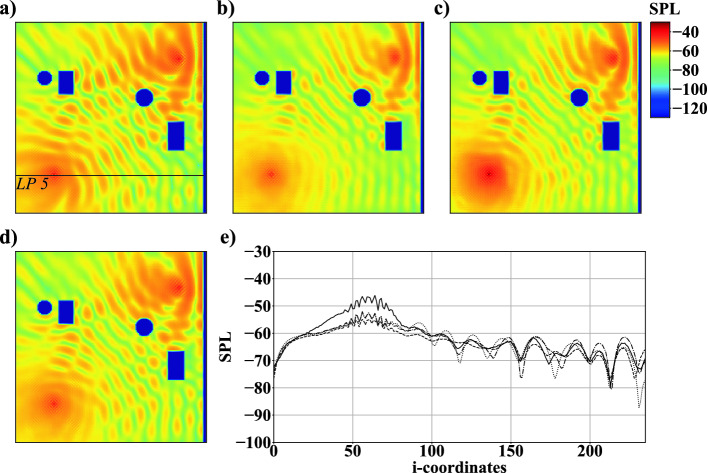
Example of *SPL* fields of case 3: a) simulation result, b) network prediction with 6, 000, c) 10, 000, and d) 20, 000 simulations; *SPL* distribution at $j=80$ along *LP*5: simulation result $\cdot \cdot \cdot$, network prediction with 6, 000 - - -, 10, 000 —, and 20, 000 simulations - $\cdot$ -.

Case 3 ties on to the findings from the previous cases to predict *SPL* fields in a domain containing objects $C_{1}$, $C_{2}$, $R_{1}$, and $R_{2}$, see Fig. 1, on $\mathcal {M}_f$. From $\mathcal {M}_c$ to $\mathcal {M}_f$ the number of trainable parameters increases from 8, 253, 089 to 8, 256, 225. NRBC and WBC boundary conditions are imposed randomly at the domain boundaries. Two monopoles $M_{1}$ and $M_{2}$ are placed inside of the domain. $M_{1}$ is located at (5*C*, 5*C*) and $M_{2}$ is positioned randomly. For the training, validation, and testing of the AFP, a total number of 20, 000 simulations is used. Results of computations with different simulation inputs are compared to the ground truth in Fig. 8. Note that the WBC is imposed at domain boundary IV, however, the complete thickness $\tilde{D}$ is not visualized in the figure. The first case uses 6, 000 simulations with a distribution of 5, 200, 400, and 400 for training, validation, and testing. The second case employs 10, 000 simulations with a distribution of 8, 800, 600, and 600 for training, validation, and testing. The last case employs all 20, 000 simulations with a distribution of 18, 000, 1, 000, and 1, 000 for training, validation, and testing. For reference, the different setups and the corresponding results are listed in Table 2. Obviously, the error $\varGamma$ decreases when the number of training data is increased. From Figs. 8(c) and (e) it is evident that the AFP trained with 8, 800 datasets overpredicts the *SPL* near $M_{1}$. In general, it can be stated that with an increasing complexity the *SPL* is more difficult to predict compared to cases 1 and 2. To be more specific, from case 1 to case 3 the error $\varGamma$ increases by one order of magnitude, i.e., it is at $\varGamma =0.01937$ in case 3. However, complex acoustic fields are reproduced. For a number of 18,000 simulation, training took 96 hours to reach a convergence of the loss function.

## Summary, Conclusions, and Outlook

A deep learning method has been developed to predict the sound pressure level distribution in two-dimensional aeroacoustic setups including multiple randomly distributed rectangular and circular objects as hard reflective surfaces and monopoles as sound sources. The deep learning method is based on an encoder-decoder convolutional neural network, which has been trained with numerical simulations based on a lattice-Boltzmann method. To analyze the accuracy of the network predictions, various learning parameters have been tuned by successively increasing the complexity of the prediction cases and by analyzing different loss functions. A network containing 8, 256, 225 trainable parameters, a combination of the mean-squared error loss and gradient loss formulated by a 2nd-order accurate central difference scheme, and a batch size of five positively influenced the predictions. A number of 18, 000 datasets has been used to train the deep neural network. A mean absolute error of less than $2\%$ shows the neural network being capable of accurately predicting the acoustic fields. The study has been complemented with a grid convergence study, which revealed that a resolution of 50 cells for a single wavelength is sufficient to yield accurate results.

At present, the method is spatially limited to two-dimensional cases. However, most engineering applications, e.g., design processes to find optimal layouts for low-noise turbojet engines, feature three-dimensional phenomena. Extending the presented deep learning method to learn from three-dimensional simulations will lead to accelerated predictions of three-dimensional aeroacoustic problems. Furthermore, realistic acoustic fields are frequently characterized by interactions of multiple noise sources with various frequencies and amplitudes. Therefore, it is necessary to extend the current setup to monopoles with multiple frequencies and amplitudes. Apart from increasing the domain’s complexity, the level of generalization will be increased. The presented acoustic field predictor has been trained and tested on similar situations. Its capabilities to generalize will be enhanced by testing on situations that have not been part of the training process, e.g., training with four objects and testing with five. Instead of strictly separating different gradient losses, the impact of combining them in a single loss and employing individual weights will be analyzed. In addition, physics-informed losses that allow the network to comply with physical laws of aeroacoustics will be integrated. Furthermore, adversarial training will be investigated by adding a discriminator with an adversarial loss to the current architecture. Such GAN type architectures have the potential to help finding a suitable loss from the training data. It is also worth mentioning that the method presented in this study has the potential to support solving noise control problems. It remains to investigate if a dedicated acoustic field predictor that can quickly give feedback on the arrangement of multiple monopoles is capable of finding optimal acoustic setups. Therefore, the presented acoustic field predictor will be integrated into a reinforcement learning loop.
